# Correlation between EASIX and short- and long-term prognosis of patients with ischemic stroke

**DOI:** 10.1186/s12883-025-04604-8

**Published:** 2025-12-31

**Authors:** Jinchuang Li, Zhouxin Wu, Zhuoshi Yang, Xiaobing Zhang, Guobao Yang, Yang Zhang, Hongyu Wang, Xiaoyu Liu, Jing Sun

**Affiliations:** 1 First School of Clinical Medicine, Heilongjiang University of Chinese Medicine, Harbin, Heilongjiang 150040 China; 2 Heilongjiang Mingshui Kangying Hospital Co., Ltd, Suihua, Heilongjiang 151700 China; 3 Hebei University of Traditional Chinese Medicine, Shijiazhuang , Hebei 050200 China; 4 Department of Cardiovascular Medicine, First Affiliated Hospital of Heilongjiang University of Traditional Chinese Medicine, Harbin, Heilongjiang 150040 China

**Keywords:** Endothelial activation and stress index (EASIX), Ischemic stroke, Prognosis, Death risk, MIMIC-IV database

## Abstract

**Background:**

This study utilized the MIMIC-IV 3.0 database to investigate the correlation between the endothelial activation and stress index (EASIX) and the short-term (30-day) and long-term (1-year) death rates of patients with ischemic stroke (IS), thus providing insights into optimizing the risk stratification and management in clinical practice.

**Methods:**

Data from the MIMIC-IV 3.0 database were used. IS patients were identified by ICD codes. log2-EASIX scores were calculated based on admission platelet count, creatinine, and lactate dehydrogenase levels and patients were grouped into quartiles. The primary outcome was 30-day all - cause death rate, and the secondary was 1-year death rate. Multivariate Cox models, LASSO regression, Kaplan - Meier curves, restricted cubic splines, subgroup and interaction analyses were performed. R software was used for data cleaning and statistical analysis.

**Results:**

This study enrolled 3,625 acute IS patients, stratified into four groups by log₂-EASIX quartiles (Q1: -3.24 to -0.55; Q2: -0.55, 0.17]; Q3: 0.17, 1.06]; Q4: 1.06 to 7.15). Q4 had markedly higher 30-day (32.0%) and 1-year (50.7%) mortality than Q1 (15.0%, 29.3%). Fully adjusted Cox models showed Q4 vs. Q1 had elevated 30-day (HR = 1.291, 95%CI:1.035–1.610, *P* = 0.024) and 1-year (HR = 1.246, 95%CI:1.059–1.467, *P* = 0.008) mortality risks, with a significant 1-year mortality trend (*P* = 0.004). RCS analysis revealed nonlinear associations between EASIX and both mortalities (all *P* < 0.05). Bonferroni-corrected subgroup analyses found only GCS had a modifying effect (*P* < 0.004). ROC analysis showed EASIX had moderate predictive value (30-day AUC = 0.7545; 1-year AUC = 0.7277).

**Conclusions:**

EASIX is independently linked to short- and medium-term ACM in ICU-admitted IS patients; higher EASIX correlates with increased mortality, serving as a useful risk stratification and prognosis tool. Limited to moderate-severe ICU IS cases, prospective studies are required to verify its causal mechanisms.

**Supplementary Information:**

The online version contains supplementary material available at 10.1186/s12883-025-04604-8.

## Background

Ischemic stroke (IS) stands as a major cause of death and permanent disability worldwide, accounting for 80% of all stroke cases. It causes huge healthcare and economic strains in low- and middle-income countries [1, 2]. IS is marked by ischemia and hypoxia due to cerebral vascular occlusion, and it can ultimately lead to neuronal necrosis. This disease is manifested by focal neurological deficits such as trigeminal neuralgia, hemiplegia, cognitive dysfunction, ataxia, and hemisensory impairment [3]. IS typically occurs acutely, reaching its peak within hours or days. Although thrombolytic therapy and endovascular interventions have notably improved survival rates in the acute phase, patients remain at high risk of severe recurrence, long-term cognitive decline, and disability [4, 5]. Moreover, ICU patients with IS often have severe disorders of consciousness and complex conditions, thus leading to a more elevated risk of death [6]. Therefore, early diagnosis, monitoring, and intervention are critical for improving the outcomes of IS patients.

In current clinical practice, early diagnostic and risk assessment tools including the National Institutes of Health Stroke Scale (NIHSS) [7], as well as imaging scoring systems, such as the Alberta Stroke Program Early CT Score (ASPECTS) and diffusion weighted imaging (DWI)-ASPECTS, can assist in evaluating the initial condition of IS patients [8]. However, these methods have notable limitations. NIHSS and Glasgow Coma Scale (GCS) scores are inherently subjective, leading to potential interobserver variability. Since the ASPECTS score depends on an early CT scan and DWI-ASPECTS requires diffusion weighted imaging, the two scoring systems necessitate specialized equipment and trained personnel. Furthermore, in the early stages of IS, ischemic regions may not be clearly visible on imaging, making accurate assessment difficult. Delayed imaging, on the other hand, cannot capture the initial state of the disease. Compared to imaging-based assessments, blood-based scoring systems have distinct advantages. Routine blood tests, which are simple to perform and can provide rapid results, are widely available at all levels of healthcare facilities. Blood panels contain multiple indicators that can reflect hematological status. Thus, scoring systems based on these indicators can effectively capture IS-related risk factors. Currently, several biomarkers related to IS prognosis have been investigated and validated, such as the triglyceride-glucose index [9] and the stress-induced hyperglycemia ratio [10, 11]. These biomarkers offer clinicians valuable insights for clinical decision-making and risk stratification. Therefore, identifying reliable biomarkers to optimize risk assessment, guide personalized treatment, and improve IS prognosis is crucial in both research and clinical practice.

Endothelial dysfunction plays a central role in IS pathophysiology as it can exacerbate brain injury by promoting thrombosis, disrupting the blood-brain barrier, and amplifying inflammatory cascades [12, 13]. The endothelial activation and stress index (EASIX) was introduced by researchers from Germany and the United States in 2017. The EASIX score is calculated as [lactate dehydrogenase (LDH, U/L) × creatinine (mg/dL)] / platelet count (10⁹/L). This index was originally developed to assess endothelial injury in hematologic disorders [14]. Recent studies have demonstrated its prognostic value in cardiovascular diseases such as coronary artery disease. Due to the availability of its parameters (LDH, creatinine, and platelet count), EASIX has been widely adopted across different medical departments [14]. However, the link between EASIX and short- and long-term death rates in IS patients remains unclear, limiting its clinical application in this area.

This study seeks to investigate the value of EASIX for evaluating disease severity and predicting short- and long-term prognosis in IS patients based on the data from the MIMIC-IV 3.0. By filling the gaps in IS biomarker research, this study may assist in promoting precision medicine in IS and provide a scientific basis for integrating EASIX into clinical practice to optimize treatment strategies and improve patient outcomes.

## Methods

### Data collection

All samples were obtained from the MIMIC-IV (3.0) database. It is a large, general-purpose database developed and managed by the Laboratory for Computational Physiology at the Massachusetts Institute of Technology. The database contains data on 364,627 unique individuals, including 546,028 hospital admissions and 94,458 ICU stays, and covers a wide range of information such as vital signs, diagnoses, demographic characteristics, medication use, laboratory test results, and clinical outcomes. The author, Jinchuang Li, completed the required training, was granted access to the dataset (ID: 13986646), and was responsible for data extraction.

**Fig. 1:**
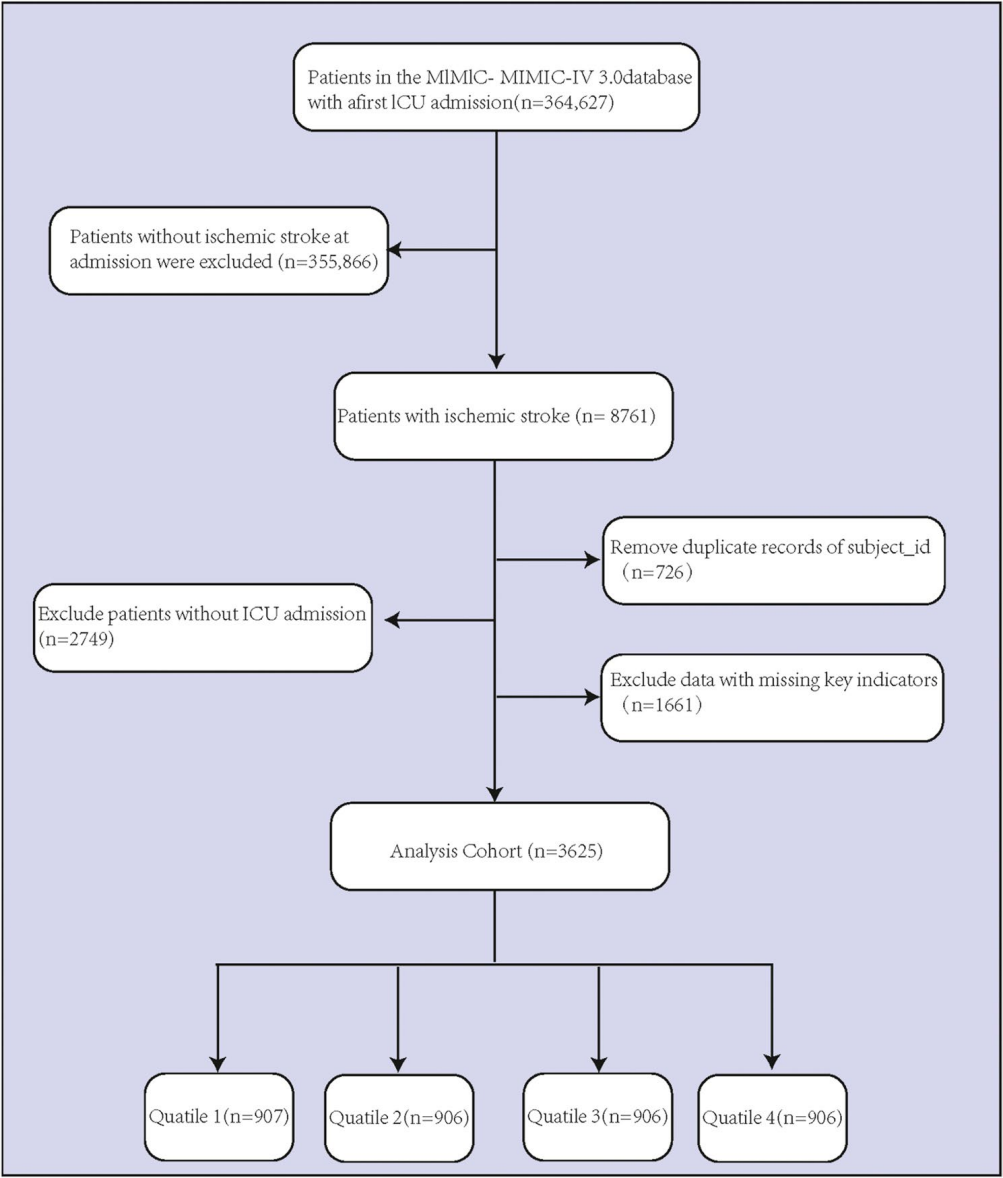
Inclusion flowchart of the study sample

This study included patients hospitalized with a primary diagnosis of acute IS. The diagnosis was based on the International Classification of Diseases, Ninth Revision (ICD-9) codes (43301, 43311, 43321, 43331, 43381, 43391, 43401, 43411, 43491) or Tenth Revision (ICD-10) code (I63). For cases involving multiple hospital admissions, only patients with the first ICU admissions specifically for acute IS were included in this study. Exclusion criteria were outlined below: (1) patients without ICU admission records; (2) patients with missing data on LDH, creatinine, or platelet count from the first hospital day; and (3) samples with no information on 30-day or 1-year survival status. The screening process for the study participants is detailed in Fig. 1.

### Data extraction

Structured query language (SQL) was applied to extract data from the MIMIC-IV database. The first recorded levels of LDH, creatinine, and platelet count upon ICU admission were used to calculate EASIX as an exposure index. The EASIX score was calculated as LDH level (U/L) × creatinine (mg/dL)/ platelet count (10⁹/L). The outcomes were all-cause 30-day and 1-year death rates following ICU admission. Other variables including (i) demographic characteristics: gender, admission age, race; (ii) vital signs: heart rate (Hr), respiratory rate (RR), peripheral oxygen saturation (SpO2); (iii) laboratory parameters: albumin, alkaline phosphatase (ALP), alanine transaminase (ALT), bilirubin, bicarbonate, calcium, chlorine, creatinine, glucose, potassium, sodium, blood urea nitrogen (BUN), lactate, creatine kinase (CK), international normalized ratio (INR), LDH, low-density lipoprotein (LDL), pH value, total cholesterol (TC), triglycerides, hemoglobin, platelet count, aspartate transaminase (AST); (iv) comorbidities: acute liver failure, acute heart failure, acute respiratory failure, sepsis, cirrhosis, diabetes mellitus, hypertension, obesity, atrial fibrillation (AF); (v) scoring systems: GCS; (vi) medications: vasopressin, proton pump inhibitors (PPI), recombinant tissue plasminogen activator (rtPA), thrombolytics; (vii) procedures/interventions: mechanical ventilation (MV), dilation, stenting, extracorporeal therapies. For all variables, only the first recorded values upon ICU admission were considered.

### Management of missing and outlier data

Data with missing key indicators (LDH, creatinine, platelet count, 30-day status) were directly deleted. Variables with a missing proportion exceeding 30% were also removed. For variables with less than 30% missing data, the multiple imputation was performed utilizing the random forest algorithm within the “mice” package in R4.4.2. The number of imputations (m) was set to 5. Variables included in the imputation model were: albumin, ALP, ALT, bilirubin, bicarbonate, calcium, chlorine, glucose, potassium, sodium, BUN, lactate, CK, INR, LDL, pH, TC, triglyceride, hemoglobin, AST, HR, RR, SpO₂, MV, GCS, simplified acute physiology score II (SAPS II), and oxford acute severity of illness score (OASIS). Winsorization was utilized to handle outliers by replacing values below the 1st percentile or above the 99th percentile.

### Statistical analysis

The Kolmogorov-Smirnov test was applied to assess the normality of continuous variables. Normally distributed data were presented as mean ± standard deviation (Mean ± SD), while non-normally distributed data were expressed as medians with interquartile ranges (IQR). For group comparisons between continuous variables, t-tests were carried out to analyze normally distributed data, and non-parametric tests were employed to analyze non-normally distributed data. Categorical variables were presented as counts and percentages (%), and group comparisons between these variables were primarily conducted via Pearson’s chi-square (χ²) test. When sample sizes were small or the expected counts were inadequate, Fisher’s exact test was applied to ensure accurate and reliable statistical results. The Least Absolute Shrinkage and Selection Operator (LASSO) regression was employed to select covariates influencing prognosis, and the selected variables were used for adjustment in multivariable analyses. Kaplan-Meier (KM) survival analysis was applied to evaluate the incidence of endpoint events based on different EASIX groups. A Cox proportional hazards (CPH) model was utilized to calculate the hazard ratio (HR) and 95% confidence interval (CI) between the EASIX score and endpoint events, and a trend test was then conducted. Moreover, multivariable analysis was carried out after adjusting for multiple potential confounders. Specifically, Model 1 represented univariate analysis without adjustment; Model 2 adjusted for general demographic factors including race, gender, and age; and Model 3 further adjusted for the variables selected by LASSO regression. Simultaneously, the proportional hazards assumption of the Cox model was systematically evaluated utilizing Schoenfeld residual tests. *P* > 0.05 indicated that the assumption was met. If any variables were found to violate the assumption, further analysis was conducted either by a stratified Cox model (stratified by the variables violating the assumption) or a Cox model with time-varying effects. Restricted cubic spline (RCS) analysis was carried out to investigate the nonlinear correlation between the EASIX and both 30-day and 1-year death risks. with the number of knots set to 5 to balance the model’s flexibility and overfitting risk. Subgroup and interaction analyses were also performed. *P* < 0.05 was considered statistically significant. In this study, 13 variables for subgroup interaction were predefined. According to the Bonferroni correction method, P for interaction less than 0.004 (= 0.05/13) was regarded as statistically significant. PostgreSQL 14.7 was applied to extract data, while data cleaning and analysis were carried out via R version 4.4.2.

## Results

### Baseline demographic and clinical characteristics

Totally 3,625 patients were included in this study. They were divided into four quartile groups based on the log₂-transformed EASIX index (log₂-EASIX) at admission (Q1 [-3.24, -0.55], Q2 (-0.55, 0.17], Q3 (0.17, 1.06], and Q4 (1.06, 7.15]; Q4 having the highest log₂-EASIX level). Q4 had a significantly higher proportion of males. Furthermore, patients in Q4 were slightly older with a wider age distribution, varied in racial composition, and possessed faster and more variable HRs and RRs. In terms of outcomes, Q4 had significantly higher rates of adverse events at both 30 days and 1 year, along with a more dispersed distribution of survival time and shorter survival time observed in some patients. Laboratory indicators revealed significantly worse liver function (e.g., ALT and AST) and renal function (e.g., serum creatinine and BUN) in Q4, suggesting more severe organ dysfunction. Regarding comorbidities, the incidence of acute hepatic failure and sepsis was significantly higher in Q4. For treatment and disease severity, Q4 required a higher proportion of multiple medications and surgical interventions, with a lower GCS score. This finding indicated more severe illness and worse consciousness levels in these patients (all *P* < 0.05) (Table 1).

**Table 1:** General demographic characteristics of the study sample

Variable	Overall(*n*= 3625)	Quartiles of EASIX				*p*
		Q1 (*n*= 907)	Q2 (*n*= 906)	Q3 (*n*= 906)	Q4 (*n* = 906)	
Gender (Male,%)	1879 (51.8)	332 (36.6)	455 (50.2)	547 (60.4)	545 (60.2)	< 0.001
Age(years)	71.04 [60.31, 80.86]	67.92 [57.13, 78.07]	71.66 [61.59, 81.59]	74.77 [64.62, 83.44]	69.84 [59.39, 79.38]	< 0.001
Race (%)						< 0.001
WHITE	2220 (61.2)	591 (65.2)	588 (64.9)	546 (60.3)	495 (54.6)	
BLACK	514 (14.2)	118 (13.0)	122 (13.5)	132 (14.6)	142 (15.7)	
ASIAN	111 (3.1)	27 (3.0)	31 (3.4)	27 (3.0)	26 (2.9)	
Other	780 (21.5)	171 (18.9)	165 (18.2)	201 (22.2)	243 (26.8)	
Mortality Status(30d)	783 (21.6)	136 (15.0)	164 (18.1)	193 (21.3)	290 (32.0)	< 0.001
Mortality Status(1year)	1347 (37.2)	266 (29.3)	278 (30.7)	344 (38.0)	459 (50.7)	< 0.001
Albumin(g/dL)	3.80 [3.30, 4.20]	3.90 [3.50, 4.30]	4.00 [3.50, 4.30]	3.80 [3.40, 4.20]	3.50 [2.90, 4.00]	< 0.001
ALP(IU/L)	79.00 [62.00, 103.00]	79.00 [63.00, 99.50]	76.00 [62.00, 94.00]	80.00 [63.00, 103.75]	83.00 [62.00, 119.00]	< 0.001
ALT(IU/L)	21.00 [15.00, 34.00]	18.00 [13.00, 27.00]	21.00 [14.00, 31.00]	22.00 [15.25, 35.00]	26.00 [16.25, 56.00]	< 0.001
Bilirubin(mg/dL)	0.50 [0.30, 0.80]	0.40 [0.30, 0.60]	0.50 [0.30, 0.70]	0.50 [0.40, 0.80]	0.60 [0.40, 1.00]	< 0.001
Bicarbonate(mEq/L)	25.00 [22.00, 27.00]	25.00 [23.00, 27.00]	25.00 [23.00, 28.00]	25.00 [22.00, 27.00]	23.00 [20.00, 26.00]	< 0.001
Calcium(mg/dL)	8.90 [8.40, 9.40]	9.10 [8.60, 9.50]	9.00 [8.60, 9.47]	8.90 [8.50, 9.40]	8.70 [8.10, 9.20]	< 0.001
Chlorine(mEq/L)	102.00 [99.00, 105.00]	102.00 [99.00, 104.00]	103.00 [100.00, 105.00]	103.00 [100.00, 105.00]	103.00 [99.00, 106.00]	< 0.001
Creatinine(mg/dL)	1.00 [0.80, 1.30]	0.80 [0.70, 0.90]	0.90 [0.80, 1.10]	1.10 [0.90, 1.30]	1.50 [1.10, 2.48]	< 0.001
Glucose(mg/dL)	120.00 [98.00, 162.00]	115.00 [97.00, 146.00]	116.00 [96.00, 157.00]	121.00 [100.00, 169.00]	128.00 [103.00, 176.00]	< 0.001
Potassium(mEq/L)	4.20 [3.90, 4.60]	4.10 [3.80, 4.50]	4.20 [3.80, 4.50]	4.20 [3.90, 4.60]	4.30 [3.90, 4.88]	< 0.001
Sodium(mEq/L)	139.00 [137.00, 141.00]	139.00 [137.00, 141.00]	139.00 [137.00, 142.00]	139.00 [137.00, 142.00]	139.00 [136.00, 141.00]	< 0.001
BUN(mg/dL)	19.00 [14.00, 27.00]	15.00 [12.00, 19.00]	18.00 [14.00, 23.00]	20.00 [15.00, 27.00]	29.00 [19.00, 46.00]	< 0.001
Lactate(IU/L)	1.60 [1.20, 2.30]	1.60 [1.20, 2.30]	1.60 [1.20, 2.20]	1.60 [1.20, 2.20]	1.80 [1.30, 2.70]	< 0.001
CK(U/L)	104.00 [57.00, 217.00]	79.00 [47.00, 143.50]	96.00 [60.00, 174.75]	112.00 [59.00, 230.75]	155.00 [74.00, 378.00]	< 0.001
INR	1.10 [1.00, 1.30]	1.10 [1.00, 1.20]	1.10 [1.00, 1.20]	1.10 [1.00, 1.30]	1.20 [1.10, 1.40]	< 0.001
LDH(IU/L)	236.00 [188.00, 338.00]	182.00 [155.00, 211.00]	213.00 [181.00, 258.75]	267.00 [216.00, 345.75]	407.50 [285.25, 660.50]	< 0.001
LDL(mg/dL)	86.00 [60.00, 113.00]	96.00 [71.50, 123.00]	91.00 [67.00, 117.00]	81.00 [59.00, 104.00]	74.00 [51.00, 103.00]	< 0.001
PH	7.39 [7.34, 7.44]	7.39 [7.35, 7.43]	7.40 [7.36, 7.44]	7.39 [7.35, 7.44]	7.37 [7.30, 7.43]	< 0.001
TC(mg/dL)	160.00 [129.00, 194.00]	174.00 [144.00, 206.50]	166.50 [137.00, 200.75]	155.00 [124.00, 186.00]	145.00 [115.00, 177.00]	< 0.001
Triglyceride(mg/dL)	119.00 [85.00, 169.00]	123.00 [86.00, 172.00]	119.50 [85.25, 165.75]	113.00 [81.00, 153.75]	125.00 [89.00, 179.00]	< 0.001
Hemoglobin(g/dL)	12.80 [11.30, 14.10]	13.00 [11.60, 14.10]	13.20 [11.80, 14.40]	12.95 [11.50, 14.20]	12.00 [10.00, 13.50]	< 0.001
Platelet(10⁹/L)	231.00 [180.00, 289.00]	295.00 [251.00, 363.00]	239.00 [203.00, 287.75]	205.50 [169.00, 252.75]	174.00 [118.25, 226.00]	< 0.001
AST(IU/L)	26.00 [19.00, 41.00]	22.00 [17.00, 28.00]	24.00 [19.00, 33.00]	28.00 [21.00, 42.00]	38.00 [23.00, 90.00]	< 0.001
Hr (bpm)	84.00 [73.00, 98.00]	84.00 [73.00, 97.00]	82.00 [72.00, 96.00]	83.00 [72.00, 96.00]	87.00 [75.00, 103.00]	< 0.001
RR	18.00 [15.00, 22.00]	18.00 [15.00, 22.00]	18.00 [15.00, 22.00]	18.00 [15.00, 22.00]	19.00 [16.00, 24.00]	< 0.001
Spo2(%)	98.00 [96.00, 100.00]	98.00 [96.00, 100.00]	98.00 [95.00, 100.00]	98.00 [96.00, 100.00]	98.00 [95.00, 100.00]	0.04
GCS	11.00 [7.00, 14.00]	13.00 [9.00, 14.00]	12.00 [7.00, 14.00]	11.00 [7.00, 14.00]	9.00 [5.00, 13.00]	< 0.001
Acute liver failure (%)	53 (1.5)	6 (0.7)	7 (0.8)	8 (0.9)	32 (3.5)	< 0.001
Acute heart failure (%)	71 (2.0)	10 (1.1)	20 (2.2)	19 (2.1)	22 (2.4)	0.18
Acute respiratory failure (%)	47 (1.3)	5 (0.6)	12 (1.3)	10 (1.1)	20 (2.2)	0.018
Sepsis (%)	715 (19.7)	131 (14.4)	158 (17.4)	177 (19.5)	249 (27.5)	< 0.001
Cirrhosis (%)	188 (5.2)	15 (1.7)	30 (3.3)	54 (6.0)	89 (9.8)	< 0.001
Diabetes (%)	1519 (41.9)	344 (37.9)	359 (39.6)	407 (44.9)	409 (45.1)	0.002
Hypertension (%)	1494 (41.2)	462 (50.9)	416 (45.9)	363 (40.1)	253 (27.9)	< 0.001
Obesity (%)	628 (17.3)	176 (19.4)	163 (18.0)	142 (15.7)	147 (16.2)	0.138
AF (%)	1677 (46.3)	353 (38.9)	406 (44.8)	485 (53.5)	433 (47.8)	< 0.001
Antiplatelet (%)	3069 (84.7)	779 (85.9)	802 (88.5)	767 (84.7)	721 (79.6)	< 0.001
Vasopressin (%)	523 (14.4)	83 (9.2)	96 (10.6)	114 (12.6)	230 (25.4)	< 0.001
PPI = Yes (%)	2185 (60.3)	522 (57.6)	545 (60.2)	528 (58.3)	590 (65.1)	0.004
rtPA (%)	827 (22.8)	181 (20.0)	172 (19.0)	196 (21.6)	278 (30.7)	< 0.001
MV (%)	1500 (41.4)	315 (34.7)	351 (38.7)	382 (42.2)	452 (49.9)	< 0.001
Dilation (%)	185 (5.1)	42 (4.6)	49 (5.4)	38 (4.2)	56 (6.2)	0.23
STENT (%)	222 (6.1)	61 (6.7)	53 (5.8)	53 (5.8)	55 (6.1)	0.847
ETHNV (%)	287 (7.9)	88 (9.7)	78 (8.6)	78 (8.6)	43 (4.7)	0.001

### LASSO regression for covariate selection

To address multicollinearity, variables with a variance inflation factor (VIF) > 5 (including LDH, creatinine, platelet count) were excluded from the model. No multicollinearity was found among the remaining variables.

**Fig. 2:**
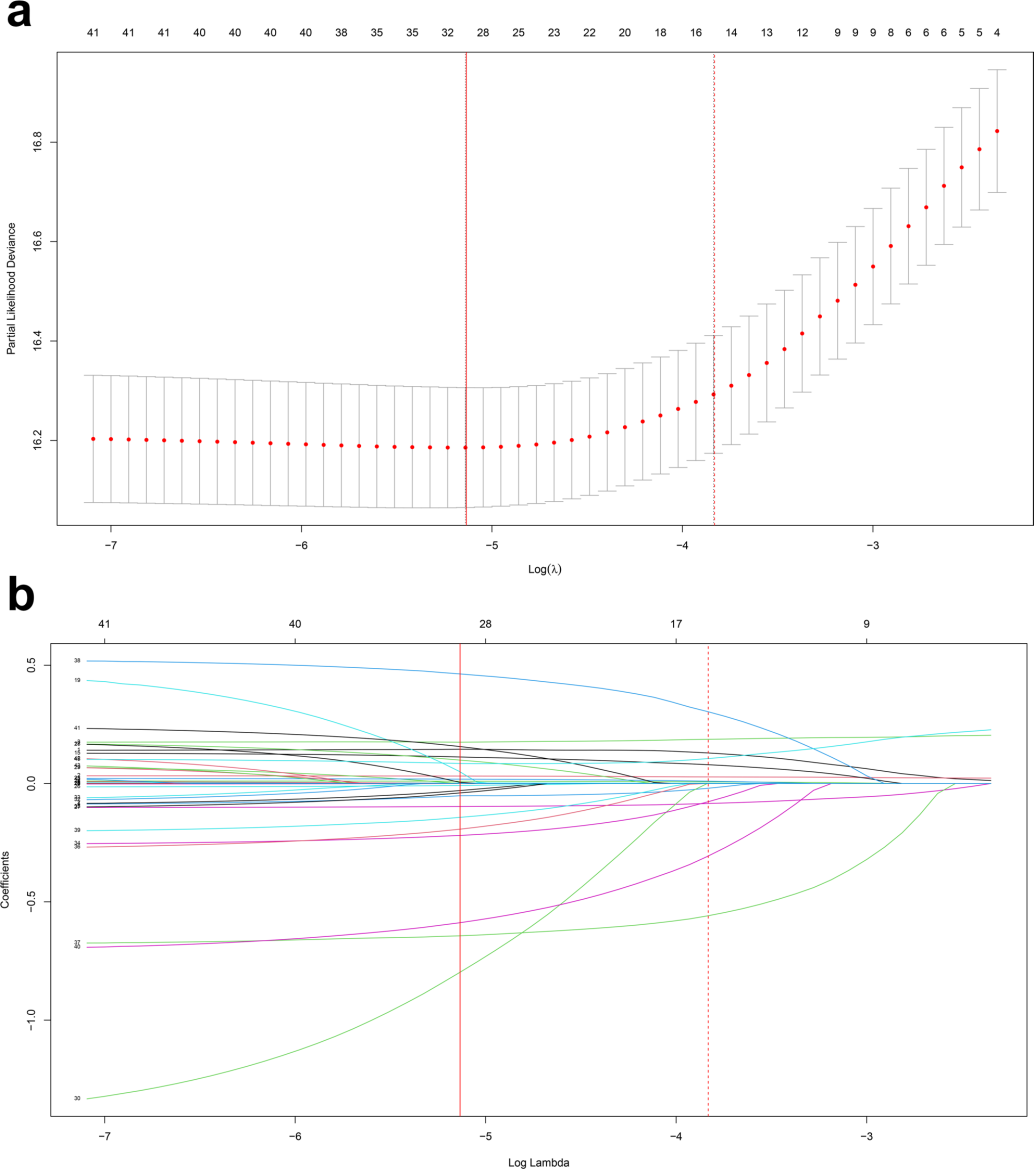
Clinical variables for 30-day outcomes selected via the LASSO regression model. Note: Figure 2**a** In LASSO regression, the optimal parameter (λ) was selected as the value with the minimum cross-validation error. The relationship between a partial likelihood deviance (binomial deviance) curve and log(λ) was plotted. By selecting the largest λ value within one standard error of the minimum error, a vertical dashed line was drawn at the optimal log(λ). Figure 2**b** LASSO coefficient trajectories for 45 covariates. A coefficient plot was generated based on the log(λ) sequence. A vertical dashed line was drawn at the optimal log(λ) value selected by cross-validation, where the optimal λ corresponded to 10 variables with nonzero coefficients

The outcome of 30-day survival was used as the dependent variable, while the log2-transformed EASIX score, demographic data, admission laboratory parameters, vital signs, comorbidities, and other relevant variables were presented as covariates. The remaining variables were included in the LASSO regression model for adjustment. 10 covariates (albumin, ALP, lactate, Hr, RR, GCS, hypertension, antiplatelets, vasopressin, and rtPA) were selected based on the optimal λ value (as illustrated in Fig. 2a and b). Additionally, by using 1-year survival data as the dependent variable, 11 covariates were selected, namely albumin, ALP, lactate, HR, RR, GCS, hypertension, antiplatelet medications, vasopressors, rtPA, and sepsis (Figures S1a and S1b). These 11 covariates were subsequently utilized in the supplementary analysis of a fully adjusted model to verify the robustness of the results.

### Kaplan-Meier survival analysis curves

**Fig. 3:**
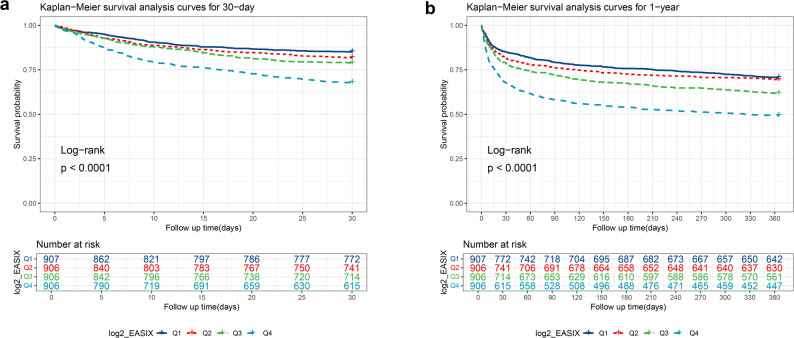
KM survival analysis curves for 30-day and 1-year mortality of patients. Note: Figure 3a 30-day survival curve. 3b 1-year survival curve. Both have log-rank P < 0.0001, and higher log₂-EASIX levels link to lower survival probabilities

The results from Fig. 3a (30-day survival curve) showed that the initial number of patients at risk for all quartile groups of log₂-EASIX (Q1-Q4) were 906, 906, 906, and 907, respectively. A statistically significant difference was identified in 30-day survival probabilities among the groups (log-rank *P* < 0.0001). At the 30-day follow-up endpoint, Q4 (with the highest log₂-EASIX) had the fewest survivors (615) and a significantly lower survival probability compared to the Q1-Q3. In contrast, Q1 (with the lowest log₂-EASIX) had the most survivors (790) and the highest survival probability. This disparity suggested that higher log₂-EASIX levels were associated with lower survival probabilities within 30 days. The results from Fig. 3b (1-year survival curve) indicated that the initial number of patients at risk for each group was consistent with that in the 30-day survival analysis. The difference in 1-year survival among the groups was still statistically significant (log-rank *P* < 0.0001). At the 360-day (1-year) follow-up, Q4 had only 476 survivors, with a significantly lower survival probability compared to the other three groups. In contrast, Q1 maintained the highest survival probability with 659 survivors. Quantitative results demonstrated that as log₂-EASIX levels increased, the survival probability of patients within one year progressively decreased, and this survival difference persisted throughout the entire follow-up period.

### Cox proportional hazards model

This study employed a multivariable Cox proportional hazards regression model to ascertain the relation between log₂-EASIX quartiles and the mortality risk of patients. Regarding 30-day mortality, univariable analysis (Model 1, unadjusted) showed that Q4 (with the highest log₂-EASIX) had a significantly greater risk of death compared to Q1 (HR = 2.391; 95% CI: 1.949–2.932; *P* < 0.001). This association remained significant after adjustments for demographic confounders (age, sex, race; Model 2) (HR = 2.300; 95% CI: 1.868–2.833; *P* < 0.001). After further adjustments for laboratory indicators, clinical symptoms, and comorbidities in the fully adjusted model (Model 3), the mortality risk for Q4 was still higher than that for Q1 (HR = 1.291; 95% CI: 1.035–1.610; *P* = 0.024). Trend tests indicated that 30-day mortality significantly increased with ascending log₂-EASIX quartiles in both the unadjusted (P for trend < 0.001) and partially adjusted models (P for trend < 0.001). However, this trend was not statistically significant in the fully adjusted model (P for trend = 0.862).

For 1-year mortality, univariable analysis (Model 1) showed that Q4 had a significantly greater risk of death compared to Q1 (HR = 2.087; 95% CI: 1.794–2.427; *P* < 0.001). After adjustments for demographic factors (Model 2), the association was still significant (HR = 2.035; 95% CI: 1.744–2.375; *P* < 0.001). In the fully adjusted model (Model 3), the mortality risk for Q4 was still higher than that for Q1 (HR = 1.246; 95% CI: 1.059–1.467; *P* = 0.008). Trend tests indicated that 1-year mortality significantly increased with ascending log₂-EASIX quartiles in the unadjusted (P for trend < 0.001), partially adjusted (P for trend < 0.001), and fully adjusted models (P for trend = 0.004) (Table 2).

**Table 2:** Cox proportional hazard ratios for 30-day and 1-year mortality

Factor	Model1			Model2			Model3		
	HR	95%CI	*P*	HR	95%CI	*P*	HR	95%CI	*P*
30-day mortality	P for trend:<0.001			P for trend:<0.001			P for trend: 0.862		
log2_EASIX quartile									
Q1[-3.24, -0.55]	—	—		—	—		—	—	
Q2[-0.55, 0.17]	1.243	0.990, 1.561	0.061	1.163	0.925, 1.463	0.196	1.087	0.863, 1.368	0.48
Q3[0.17, 1.06]	1.469	1.179, 1.831	< 0.001	1.299	1.037, 1.628	0.023	1.019	0.812, 1.280	0.869
Q4[1.06, 7.15]	2.391	1.949, 2.932	< 0.001	2.3	1.868, 2.833	< 0.001	1.291	1.035, 1.610	0.024
1-year mortality	P for trend: <0.001			P for trend: <0.001			P for trend: 0.004		
log2_EASIX quartile									
Q1[-3.24, -0.55]	—	—		—	—		—	—	
Q2[-0.55, 0.17]	1.071	0.905, 1.267	0.427	0.991	0.837, 1.175	0.92	0.941	0.794, 1.116	0.45
Q3[0.17, 1.06]	1.385	1.180, 1.625	< 0.001	1.218	1.033, 1.436	0.019	0.989	0.838, 1.167	0.874
Q4[1.06, 7.15]	2.087	1.794, 2.427	< 0.001	2.035	1.744, 2.375	< 0.001	1.246	1.059, 1.467	0.008

Model 1: unadjusted

Model 2: adjusted foradmission_age, gender, race

Model 3: adjusted for admission_age, gender, race, albumin, alp, lactate, hr, rr, gcs, hypertension, use of antiplatelet/vasopressin, rtpa use

To further evaluate the robustness of the results, the Schoenfeld residual test was performed. The robust results (*P* > 0.05) were observed (Figures S2a to S2f). Additionally, a supplementary Cox regression was conducted utilizing the 11 covariates selected by LASSO regression with one-year survival data as the dependent variable for full adjustments. The results were still significant (Table S1).

### Restricted cubic spline regression analysis

**Fig. 4:**
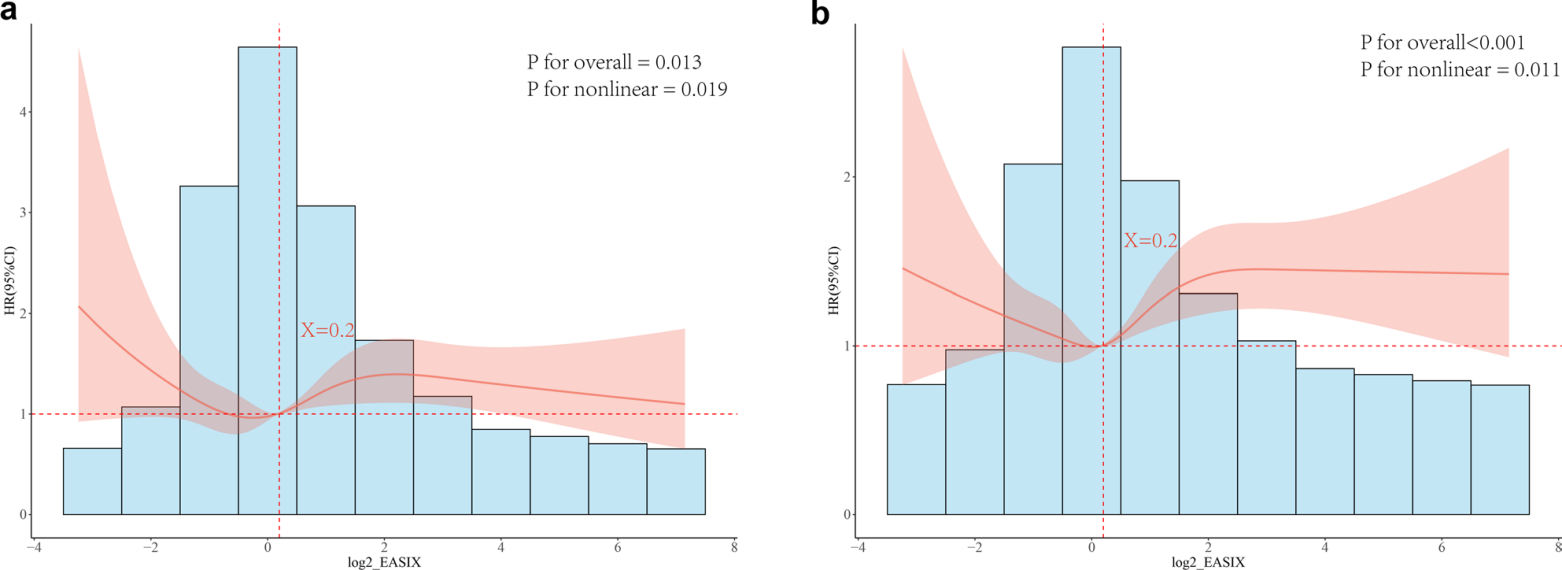
RCS curves for 30-day and 1-year mortality. Note: 4a RCS for 30-day mortality: overall association P=0.013, nonlinear association P=0.019. 4b RCS for 1-year mortality: overall association P<0.001, nonlinear association P=0.011. The dashed line marks the reference point (X=0.2, log₂-EASIX); shaded areas represent 95% confidence intervals, and bars indicate the distribution of log₂-EASIX values

The results in Fig. 4a showed that within the range of -2 to 8 for log₂-EASIX, the association of log₂-EASIX with 30-day mortality was statistically significant (overall *P* = 0.013) and was substantially nonlinear (P for nonlinearity = 0.019). With log₂-EASIX = 0.2 as the reference point, HR values fluctuated around 1.0 when log₂-EASIX was below this point. This feature suggested a weak association between log₂-EASIX levels and 30-day mortality risk in this interval. When log₂-EASIX exceeded the reference point (particularly after reaching ≥ 2), HR values progressively increased to approximately 3.0 at the maximum. This trend indicated that higher log₂-EASIX levels were nonlinearly associated with the elevated 30-day mortality risk. The results in Fig. 4b demonstrated that the overall association between log₂-EASIX and 1-year mortality was highly statistically significant (overall *P* < 0.001) and substantially nonlinear (P for nonlinearity = 0.011). With log₂-EASIX = 0.2 as the reference point (HR = 1.0), HR values remained near 1.0 when log₂-EASIX was below this point, indicating no substantial fluctuation in risk. Once log₂-EASIX exceeded the reference point, HR values gradually increased with rising log₂-EASIX levels. This trend suggested that during the long-term follow-up, the nonlinear effect of higher log₂-EASIX levels on mortality risk persisted, and the strength of this association was greater than that observed in the short-term follow-up.

### Subgroup analysis and forest plot

**Fig. 5:**
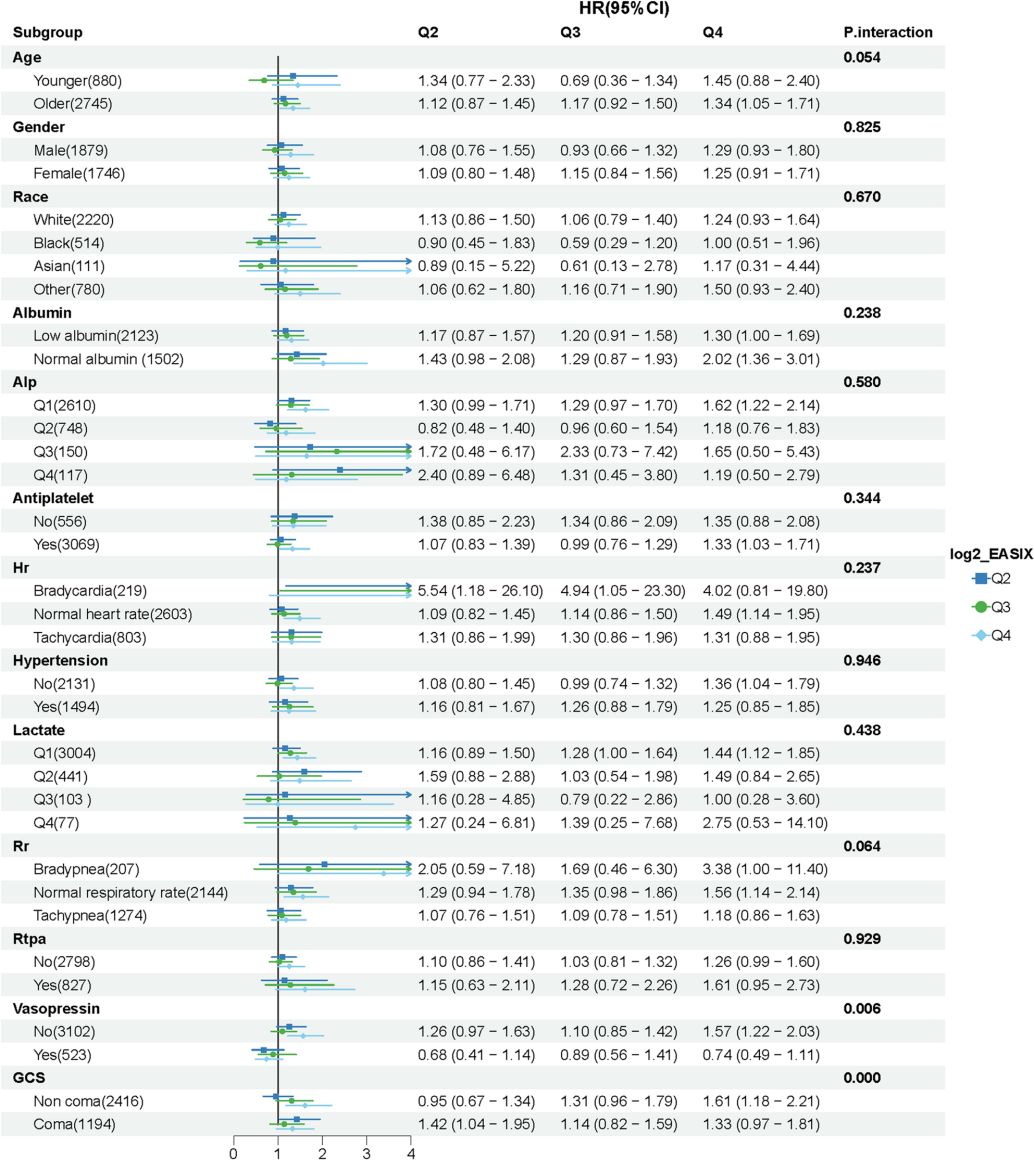
Forest plot of HR for mortality across different subgroups. Note: GCS: 3–8: Coma, >8: Non-coma. Age: ≥60 years: Older, <60 years: Younger Albumin levels: Low serum albumin (<4 g/dL); normal serum albumin (≥4 g/dL). ALP & Lactate: Categorized into quartiles. Hr: 60–100 bpm: Normal; >100 bpm: Tachycardia; <60 bpm: Bradycardia. RR: 12–20 breaths/min: Normal; >20: Tachypnea; <12: Bradypnea

This study examined 13 predefined subgroup interaction variables. The Bonferroni correction method was applied to control multiple testing errors, and *P* < 0.004 (0.05/13) served as the threshold for statistical significance of interactions. Subgroup consistency in the association between Q2-Q4 and mortality risk was analyzed based on the fully adjusted Cox model (with log₂-EASIX Q1 as the reference). The results showed that only the GCS grade subgroup had a significant interaction effect (*P* = 0.000 < 0.004). Within the non-coma subgroup (2,416), patients in Q4 had an HR of 1.61 (95% CI: 1.18–2.21). In contrast, within the coma subgroup (1,194), patients in Q4 had an HR of 1.33 (95% CI: 0.97–1.81). These results suggested that the consciousness status of patients significantly modified the predictive utility of log₂-EASIX for mortality risk. For the remaining 12 subgroups, including demographic characteristics (age, sex, and race), laboratory indicators (albumin, ALP, and lactate), vital signs (HR and RR), and treatments (antiplatelet medications, rtPA, and vasopressin), none of the P-values for interaction reached the correction standard (e.g., sex: *P* = 0.825; race: *P* = 0.670; albumin: *P* = 0.238; vasopressin utilization: *P* = 0.006; for vasopressin utilization, *P* < 0.05 was observed before correction but the P-value was not significant after correction). Among these subgroups, Q4 generally showed an elevated trend in mortality risk, and the overall association between log₂-EASIX and mortality risk remained consistent without significant modification by the respective factors (Fig. 5).

### Receiver Operating Characteristic (ROC) curve analysis

**Fig. 6:**
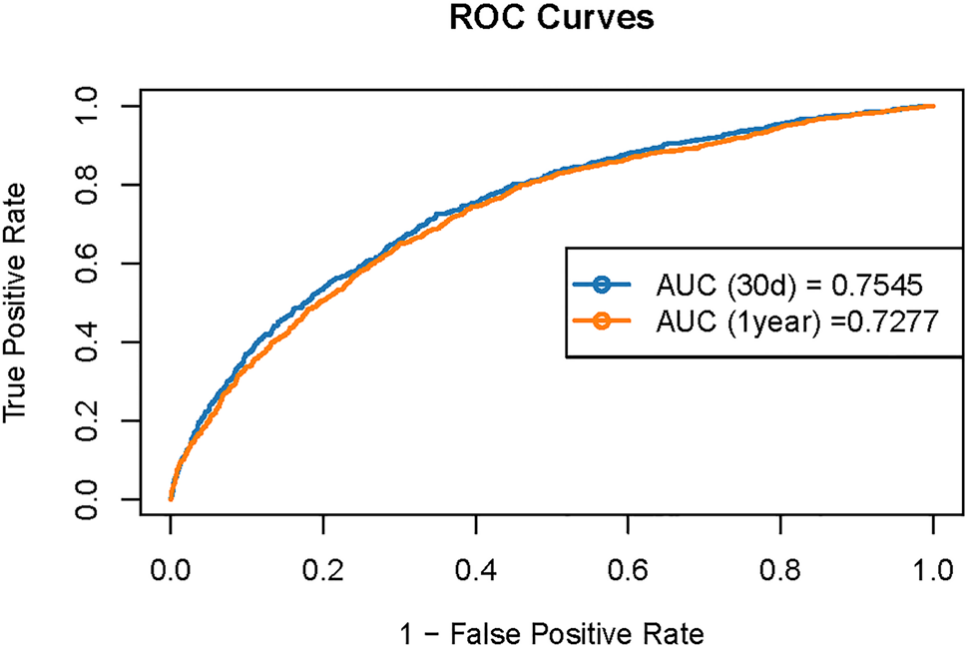
ROC curves of the EASIX prediction model for 30-ay and 1-year prognosis in patients with cerebral infarction

The ROC curve analysis was implemented for log₂-EASIX for predicting prognosis in patients with acute IS under Model 3 (the fully adjusted model). The results showed that, in this model, the area under the curve (AUC) for log₂-EASIX for predicting 30-day mortality was 0.7545, and the AUC for log₂-EASIX for predicting 1-year mortality was 0.7277. This finding indicated that after full adjustments for potential confounders, log₂-EASIX still had moderate predictive performance for both short-term (30-day) and medium-term (1-year) mortality risk in this population (Fig. 6).

## Discussion

This study analyzed 3625 IS patients from the MIMIC-IV (3.0) database to investigate the link between the EASIX and short- and long-term prognosis. The findings suggest that EASIX is a valuable prognostic marker in IS, indicating a positive correlation between 30-day and 1-year death risks.

The core pathology of IS is ischemia and hypoxia caused by vascular occlusion, and endothelial dysfunction is present throughout the disease course. Studies have linked IS to elevated levels of endothelial activation in the systemic circulation [15]. The endothelium regulates vascular homeostasis by producing and releasing cytokines and chemical mediators. Thus, it can serve as an independent risk factor for IS [16]. During ischemia, structural and functional changes in cerebral endothelial cells lead to blood-brain barrier disruption, vascular inflammation, edema, and angiogenesis. These complex pathological processes directly cause IS, neurological deficits, and post-stroke neurovascular remodeling [17]. Moreover, the severity of endothelial dysfunction is linked to IS prognosis [18].

The endothelium, a single layer of cells that line the blood vessels, acts not only as a physical barrier but also as a key regulator of vascular function [19]. Endothelial dysfunction is linked to increased LDH levels produced by endothelial cells. It may caused by the direct release of LDH from endothelial cells and circulating cells such as thrombocytes and leukocytes [20–22]. Creatinine levels, a marker of renal function, indirectly reflect microcirculatory disorders and ineffective tissue perfusion. Endothelial dysfunction often manifests as renal impairment (elevated creatinine levels). Endothelial cells can maintain vascular integrity by regulating metabolism and platelet activation [23]. Additionally, low platelet counts may be partly caused by endothelial injury and complement activation in various diseases [24]. Notably, researchers have confirmed that EASIX correlates with biomarkers of endothelial activation, such as chemokine (C-X-C motif) ligand 8 and insulin-like growth factor-1 serum levels [25]. These findings support the role of EASIX as a marker of systemic endothelial dysfunction.

Since the EASIX is easily accessible and cost-effective in clinical practice, it can be used to assess the risk of complications and prognosis. Previous studies have validated EASIX as a biomarker for predicting endothelial dysfunction and survival rates in patients with graft-versus-host disease (GVHD) [25, 26]. Moreover, EASIX has been recognized as a practical biomarker in multiple myeloma [27], COVID-19 [28], small-cell lung cancer [29], sepsis [24], critically ill patients with advanced liver disease [30], traumatic brain injury [31], and sinusoidal obstruction syndrome [32]. These findings reinforce the clinical utility of EASIX for predicting IS prognosis. However, it must be acknowledged that EASIX may reflect the overall severity of critical illness rather than solely stroke-specific endothelial injury.

Totally 3,625 ICU-admitted IS patients were included in this study. Subgroup analyses were implemented utilizing the fully adjusted Cox model. The results showed that the association between EASIX and IS prognosis remained consistent across all 13 predefined subgroups (including sex, race, albumin, and hypertension) and did not reach the significant standard for Bonferroni correction (*P* > 0.004), highlighting the robustness of this association. Furthermore, before correction, EASIX interacted with HR, vasopressin utilization, and GCS grade (*P* < 0.05). After correction, however, only the GCS grade significantly modified the association. Specifically, within the non-coma subgroup (GCS > 8), patients in Q4 (log₂-EASIX: 1.06–7.15) had a higher mortality risk (HR = 1.61) than that within the coma subgroup (GCS 3–8, HR = 1.33).

Furthermore, in subgroups including old patients, individuals with normal albumin levels, normal HRs/RRs, and non-users of vasopressin, the predictive performance of EASIX for all-cause mortality risk may be more superior. Within Q4 (with the highest EASIX quartile), while the lactate subgroup had an increased mortality risk, the estimates were associated with greater uncertainty. Additionally, patients with normal albumin levels in Q4 had a significantly higher mortality risk. The mechanisms underlying these specific associations remain unclear and should be further validated in subsequent studies.

This study has several limitations. (a) First, based on a retrospective database analysis, it may have selection bias. The deletion strategy for missing data adopted in this study aims to preserve the authenticity of EASIX and maintain an adequate sample size, thus keeping the overall risk of selection bias manageable. Nevertheless, the bias due to non-random missingness should be considered cautiously. The likely direction of such bias may lead to an underestimation of the independent predictive utility of EASIX, potentially distorting the adjustment effects of covariates in the model and confounding the true association between EASIX and the outcomes. (b) Additionally, patients in Q4 were old and had significantly higher incidences of sepsis, acute liver failure, and acute respiratory failure compared to Q1. This pattern suggests that the association between EASIX and prognosis may be disturbed by confounding effects of critical illness. From the perspective of pathological mechanisms, conditions like sepsis and multi-organ dysfunction can significantly elevate levels of LDH (a marker of cellular injury) and creatinine (a marker of renal impairment), and reduce platelet counts (due to consumptive processes). (c) Despite multivariable adjustments performed in this study, the influence of residual confounding cannot be entirely ruled out. The primary reason is that certain key variables, which are associated with both EASIX and the prognosis of IS, were omitted due to missing information in the database or not being included in the analysis. (d) The conclusions of this study are only applicable to “moderate to severe IS patients admitted to the ICU.” Future research should validate the distribution of EASIX and its association with prognosis in non-ICU or mild IS cohorts and adapt EASIX for predicting clinical endpoints in mild cases to provide a simple risk stratification tool for basic medical institutions. (e) Limited by the retrospective database, some ideal covariates were unavailable. Future prospective studies should incorporate relevant additional variables. (f) Furthermore, while EASIX is associated with mortality risk, its specific causal role in the pathogenesis of IS cannot be determined in this study. Further basic and prospective investigations are needed to delve into this relationship.

## Conclusion

This study identified EASIX as an effective predictor of short-term (30 day) and long-term (1year) death rates in IS patients. Patients with higher EASIX scores (Q4) had significantly increased risks of death, suggesting that endothelial injury may play a key role in IS prognosis.

## Supplementary Information

Supplementary Material 1: Table S1 Cox proportional hazard ratios for 30-day and 1-year mortality.

Supplementary Material 2: Figure S1 Clinical variables for 1-year outcomes selected via the LASSO regression model. Note: Eleven covariates (albumin, ALP, lactate, HR, RR, GCS, hypertension, antiplatelet medications, vasopressors, rtPA, and sepsis) were selected via LASSO regression with 1-year survival as the dependent variable.

Supplementary Material 3: Figure S2 Schoenfeld residual test plots for validating the Cox proportional hazards assumption of log₂-EASIX associated with 30-day and 1-year mortality.

## Data Availability

The data supporting the findings of this study are publicly available from the MIMIC-IV (3.0) database, which can be accessed at [https://physionet.org/content/mimiciv/3.0/](https:/physionet.org/content/mimiciv/3.0) . All relevant data used in this study are derived from this publicly accessible database, and the access complies with the data use agreement of the database.
