# Supplementary material for: Correlation between EASIX and short- and long-term prognosis of patients with ischemic stroke
Source: BMC Neurol. 2025 Dec 31;26:62. doi: 10.1186/s12883-025-04604-8 (PMC12866349; doi:10.1186/s12883-025-04604-8)
Supplement: Supplementary file 1 — Supplementary Material 1: Table S1 Cox proportional hazard ratios for 30-day and 1-year mortality. [file 12883_2025_4604_MOESM1_ESM.docx]

Table S1 Cox proportional hazard ratios for 30-day and 1-year mortality

| **Factor** | **Model1** | | | **Model2** | | | **Model3** | | |
| --- | --- | --- | --- | --- | --- | --- | --- | --- | --- |
|  | **HR** | **95%CI** | **P** | **HR** | **95%CI** | **P** | **HR** | **95%CI** | **P** |
| 30-days mortality | P for trend:<0.001 | | | P for trend:<0.001 | | | P for trend: 0.862 | | |
| log2_EASIX quartile |  |  |  |  |  |  |  |  |  |
| Q1[-3.24, -0.55] | — | — |  | — | — |  | — | — |  |
| Q2[-0.55, 0.17] | 1.243 | 0.990, 1.561 | 0.061 | 1.163 | 0.925, 1.463 | 0.196 | 1.087 | 0.863, 1.368 | 0.48 |
| Q3[0.17, 1.06] | 1.469 | 1.179, 1.831 | <0.001 | 1.299 | 1.037, 1.628 | 0.023 | 1.019 | 0.812, 1.280 | 0.869 |
| Q4[1.06, 7.15] | 2.391 | 1.949, 2.932 | <0.001 | 2.3 | 1.868, 2.833 | <0.001 | 1.291 | 1.035, 1.610 | 0.024 |
| 1-year mortality | P for trend:<0.001 | | | P for trend:<0.001 | | | P for trend: 0.004 | | |
| log2_EASIX quartile |  |  |  |  |  |  |  |  |  |
| Q1[-3.24, -0.55] | — | — |  | — | — |  | — | — |  |
| Q2[-0.55, 0.17] | 1.071 | 0.905, 1.267 | 0.427 | 0.991 | 0.837, 1.175 | 0.92 | 0.941 | 0.794, 1.116 | 0.45 |
| Q3[0.17, 1.06] | 1.385 | 1.180, 1.625 | <0.001 | 1.218 | 1.033, 1.436 | 0.019 | 0.989 | 0.838, 1.167 | 0.874 |
| Q4[1.06, 7.15] | 2.087 | 1.794, 2.427 | <0.001 | 2.035 | 1.744, 2.375 | <0.001 | 1.246 | 1.059, 1.467 | 0.008 |

Model 1: unadjusted

Model 2: adjusted foradmission _ age, gender, race

Model 3: adjusted for admission _ age, gender, race, albumin, alp, lactate, hr, rr, gcs, hypertension, use of antiplatelet/vasopressin, rtpa use
