# Supplementary figures and images for: Correlation between EASIX and short- and long-term prognosis of patients with ischemic stroke
Source: BMC Neurol. 2025 Dec 31;26:62. doi: 10.1186/s12883-025-04604-8 (PMC12866349; doi:10.1186/s12883-025-04604-8)

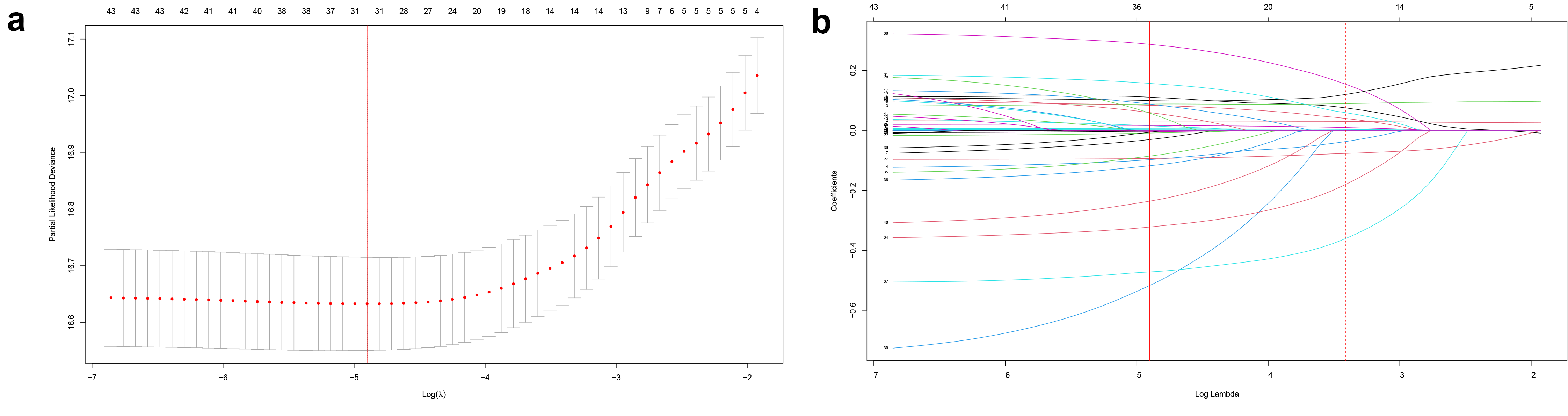

Supplement: Supplementary file 2 — Supplementary Material 2: Figure S1 Clinical variables for 1-year outcomes selected via the LASSO regression model. Note: Eleven covariates (albumin, ALP, lactate, HR, RR, GCS, hypertension, antiplatelet medications, vasopressors, rtPA, and sepsis) were selected via LASSO regression with 1-year survival as the dependent variable. [file 12883_2025_4604_MOESM2_ESM.tif]

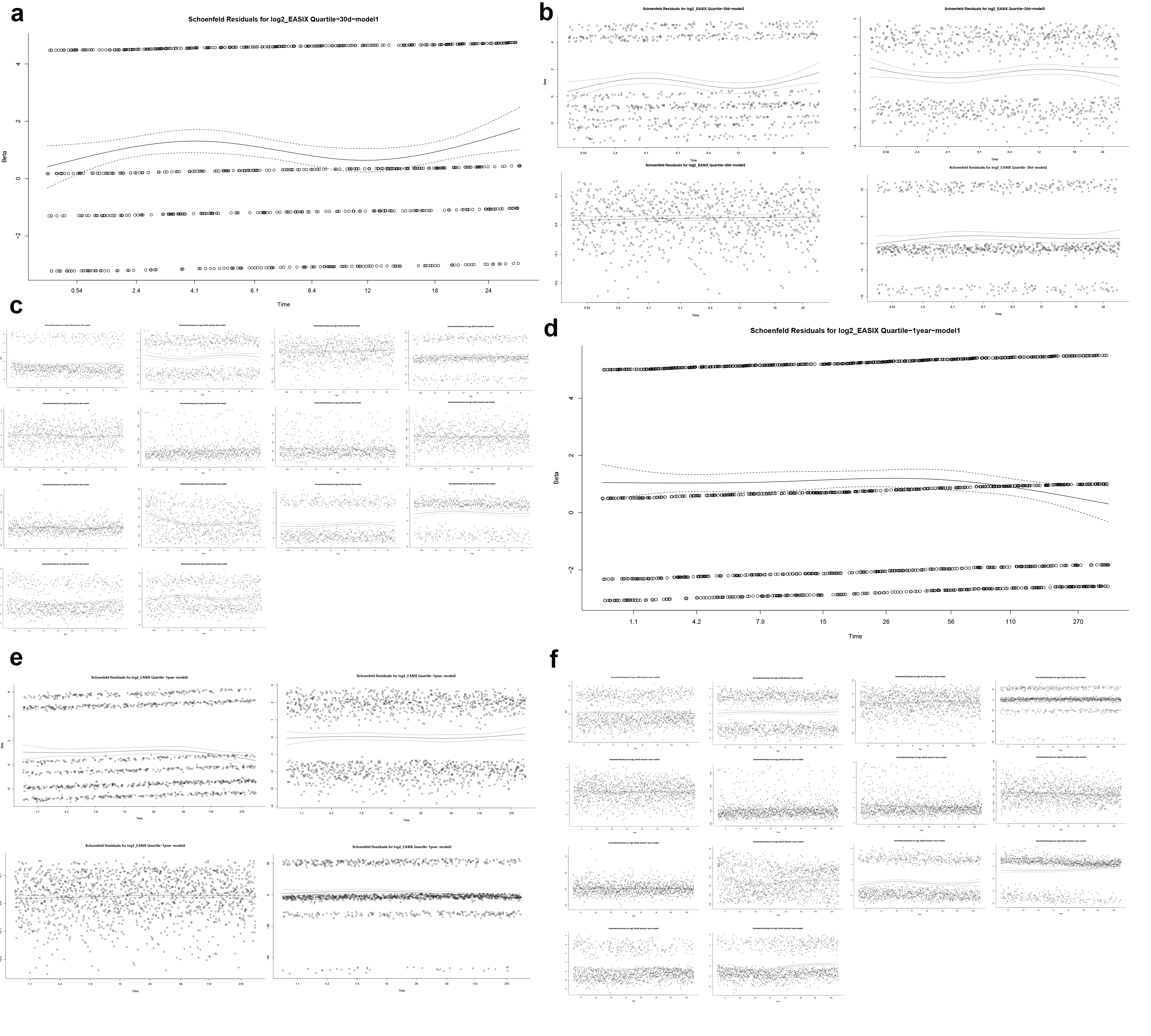

Supplement: Supplementary file 3 — Supplementary Material 3: Figure S2 Schoenfeld residual test plots for validating the Cox proportional hazards assumption of log₂-EASIX associated with 30-day and 1-year mortality. [file 12883_2025_4604_MOESM3_ESM.tif]
